# Improved accuracy for noncoplanar radiotherapy: an EPID‐based method for submillimeter alignment of linear accelerator table rotation with MV isocenter

**DOI:** 10.1120/jacmp.v15i2.4682

**Published:** 2014-03-06

**Authors:** Matthew J. Nyflot, Ning Cao, Juergen Meyer, Eric C. Ford

**Affiliations:** ^1^ Department of Radiation Oncology University of Washington Seattle WA USA

**Keywords:** treatment table, rotation, isocenter, coincidence, electronic portal imaging device (EPID)

## Abstract

Accurate alignment of linear accelerator table rotational axis with radiation isocenter is critical for noncoplanar radiotherapy applications. The purpose of the present study is to develop a method to align the table rotation axis and the MV isocenter to submillimeter accuracy. We developed a computerized method using electronic portal imaging device (EPID) and measured alignment stability over time. Mechanical and radiation isocenter coincidence was measured by placing a steel ball bearing at radiation isocenter using existing EPID techniques. Then, EPID images were acquired over the range of table rotation. A MATLAB script was developed to calculate the center of rotation, as well as the necessary adjustment to move the table rotational axis to MV isocenter. Adjustment was applied via torque to screws at the base of the linac table. Stability of rotational alignment was measured with 49 measurements over 363 days on four linacs. Initial rotational misalignment from radiation isocenter ranged from 0.91−2.11 mm on the four tested linacs. Linac‐A had greatest error (>2 mm) and was adjusted with the described method. After adjustment, the error was significantly decreased to 0.40±0.12 mm. The adjustment was stable over the course of 15 measurements over 231 days. Linac‐B was not adjusted, but tracked from time of commissioning with 27 measurements over 363 days. No discernible shift in couch characteristics was observed (mean error 1.40±0.22 mm). The greater variability for Linac‐B may relate to the interchangeable two‐piece couch, which allows more lateral movement than the one‐piece Linac‐A couch. Submillimeter isocenter alignment was achieved by applying a precision correction to the linac table base. Table rotational characteristics were shown to be stable over the course of twelve months. The accuracy and efficiency of this method may make it suitable for acceptance testing, annual quality assurance, or commissioning of highly‐conformal noncoplanar radiotherapy programs.

PACS number: 87

## I. INTRODUCTION

With the increased use of high‐dose highly conformal treatments such as stereotactic body radiotherapy, accuracy of mechanical and radiation isocenters is paramount.^(1)^ Historically, such accuracy was achieved through mechanical coupling of the patient to a frame, such as the skull‐anchored radiosurgery frames or bite‐block‐coupled floor stands.^(2)^ In recent years, stereotactic techniques are increasingly implemented with frameless methods, which permit stereotactic treatments to sites not amenable to mechanical coupling, but introduce additional isocenter uncertainty.^(3)^ Recently, highly noncoplanar techniques have been proposed to provide further dosimetric advantages through increased degrees of freedom for treatment planning.^4^, ^5^, ^6^ Accurate table rotation about radiation isocenter is an important element of conformal therapy, and critically so for noncoplanar methods. Consequently, there is a need for an accurate and efficient method to measure and adjust table rotational isocenter relative to radiation isocenter.

With QA tools typically available today it is difficult to assess the treatment table rotational isocenter with submillimeter precision. The standard test for measuring rotational alignment is the “star‐shot” test where two axes are held constant while a slit field is delivered under various rotations of the remaining axis. However, the standard table star‐shot test provides alignment information only in a single radiation plane, whereas radiation isocenter is best defined over the range of all possible gantry and collimator angles. Thus, star‐shot methods do not provide information on the alignment between the table rotational axis and the true radiation isocenter. Another method to assess the rotational alignment of the table is visual inspection of crosshair walkout on graph paper. This walkout is difficult to assess in two dimensions to less than a millimeter, even ignoring possible crosshair misalignment. The Winston‐Lutz test^(7)^ is commonly used to sample combined isocentric accuracy at relevant treatment parameters, but does not intuitively indicate the source of misalignment.

Various semiautomatic tests have been proposed for checking the alignment of linear accelerators. Mechanical methods utilizing a platform for laser alignment and a computer‐interfaced microscope have been used.^8^, ^9^ Several authors have published on computerized analysis for film star‐shots,^10^, ^11^ including correlation of film star‐shots with optical methods.^12^, ^13^ In another study, phosphor plates were used to acquire quantitative data on correlation of laser alignment with mechanical isocenter.^(14)^ Methods utilizing electronic portal imaging dosimetry (EPID) for computerized star‐shots or Winston‐Lutz analysis are in the literature,^15^, ^16^, ^17^ and these have the advantage of not requiring external systems or film.

This report suggests a complete procedure for couch alignment of Elekta accelerators that utilizes a radiation isocenter identification method from the literature,^(18)^ as well as manufacturer specifications for the adjustment of linear accelerator tables.^(19)^ This report differs from previous studies in several important ways: 1) while previous methods provide a means of measuring “wobble” as linac components are rotated, the method presented here also links the wobble to the absolute position of the radiation isocenter in space; 2) the method presented here uses a direct radiographic measure of radiation isocenter rather than relying on a surrogate for radiation isocenter such as room lasers; 3) the method presented here provides a formalism for the mechanical adjustments required to bring linac components into alignment; 4) data are presented which demonstrates correction of a misaligned table with the proposed method, as well as a year's worth of data on the stability of table characteristics, one of which was tracked from the time of commissioning. The accuracy and efficiency of this method may make it suitable for acceptance testing, annual quality assurance, and ideally suited for submillimeter alignment of the treatment table for noncoplanar radiotherapy and other precision radiotherapy applications.

## II. MATERIALS AND METHODS

**Figure 1:**
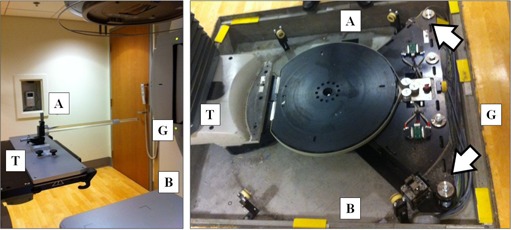
BB phantom (left) mounted on linac table. The BB is placed within 0.1 mm of megavoltage radiation isocenter prior to the 0° acquisition. Table correction (right) is applied to two screws at the table base, as indicated by arrows. Directions are noted for gun (G), target (T), A, and B sides.

The methodology described here relies on a precise knowledge of the radiation isocenter. In this report we use the term “radiation isocenter” to refer to that point from which the treatment collimator angles. This is related to, but different from, the definitions of mechanical isocenter, laser‐defined isocenter or IGRT‐derived isocenter, though the location of these points must be within the tolerances specified in TG‐142.^(1)^ To identify radiation isocenter, the first step is to align a radio‐opaque object at the point closest to treatment beam axis over many gantry and collimator positions. To accomplish this, an 8 mm steel ball bearing (BB) mounted in a Lucite rod (Elekta Synergy Basic Calibration Kit, MRT 9551; Elekta, Stockholm, Sweden) (Fig. 1) is placed at radiation isocenter using an existing digital star‐shot methodology that is provided by the vendor.^(19)^ In this method, the phantom is initially aligned to treatment lasers and eight EPID images are acquired (four cardinal gantry angles with 0° and 180° collimator angles at each gantry position). The two collimator angles are acquired in order to eliminate the effect of any asymmetry in the jaws or MLCs. For each image, the vector difference between the center of the BB and the beam axis is calculated. This information is used to calculate the correction required to minimize the displacement of the BB and the beam axis. The correction is applied via three orthogonal Vernier calipers at the base of the phantom. By iterating this process, the residual error in BB placement from radiation isocenter was within 0.11±0.05 mm over 49 measurements.

**Figure 2:**
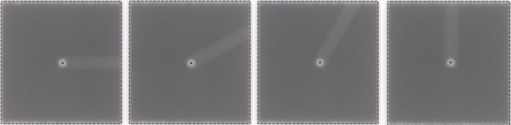
MV image of BB phantom at 0°, 30°, 60°, and 90° rotation, with BB centroid fit indicated with black dot. 15 images are acquired to map out table rotational axis.

Once the BB is positioned at the radiation isocenter using the above technique, the gantry is placed at 0° with a precision level. 15 EPID images are acquired over the range of table motion ($0^{\circ },30^{\circ },60^{\circ },90^{\circ },60^{\circ },30^{\circ },0^{\circ },-30^{\circ },-60^{\circ },90^{\circ },90^{\circ },60^{\circ },-30^{\circ },0^{\circ }$; Fig. 2) using remotely engaged table motion. The data were acquired counterclockwise and clockwise, since the trajectory was observed to take a bifurcated path or loop in some datasets.

**Figure 3:**
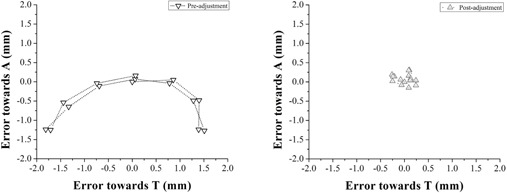
Typical plot of BB centroid displacement from isocenter at 15 table angles for Linac‐A before (left panel) and after adjustment (right panel). The calculated adjustment to move the table to radiation isocenter was −1.33 mm towards A and −0.04 mm towards T.

The resulting 15 images are exported to a MATLAB R2010b (The MathWorks, Inc., Natick, MA) program written in‐house. First, the centroid of the BB in each image is fit by first segmenting the field and BB edge and then employing centroid‐finding routines using a least squares method. The centroid fit is constrained to the projected size of the BB in the imaging plane. The BB coordinates in the initial image, which represent radiation isocenter, are defined as the (0,0) coordinate in the MV reference frame. The BB location in the following images sweeps out an arc which represents the precession of the BB about the rotational axis of the table (Fig. 3). For an idealized table, with its rotation axis exactly aligned to the radiation isocenter, the BB location will not change as the table is rotated and the 15 points will all be at (0,0) in the MV reference frame. In an actual table, the beam axis is always slightly misaligned and, therefore, the BB traces out a semicircle. The maximum rotational error is defined as the maximum vector error found at any table position. By fitting a circle to the acquired BB locations, the vector between the apparent centroid and (0,0) may be calculated. This fit was accomplished with a variant of the least‐squares Kasa method in MATLAB. The resulting vector represents the optimal adjustment to align the table rotation axis with the radiation isocenter.

Once the adjustment to radiation isocenter has been calculated it can be applied to the table base. For the linear accelerators tested here (Elekta Inc., Crawley, UK), the table rotational axis is applied by adjustment of the two base fixing screws at the table base by the service engineers (Fig. 1). By rotating the screws in the same direction, the rotational axis can be moved in the gun‐target (GT) plane. Conversely, by adjusting the screws in opposite directions, the rotational axis can be moved in the transverse (AB) plane. Care must be taken to ensure that the same torque exists on the fixings before and after adjustment. With knowledge of the accelerator geometry, an analytical expression exists to translate the adjustment vector described above to a rotational adjustment to the screws at the table base.^(19)^ This expression is described in Eq. (1) (degrees counterclockwise adjustment to A‐side fixing) and Eq. (2) (degrees clockwise adjustment to B‐side fixing), where $X_{\text{GT}}$ and $Y_{\text{AB}}$ are the prescribed shift in the GT and AB planes in millimeters:

(1) $$\theta_{A}=X_{\text{GT}}/0.01779+Y_{\text{AB}}/0.01843$$

(2) $$\theta_{B}=X_{\text{GT}}/0.01779‐Y_{\text{AB}}/0.01843$$

Reproducibility of the method was tested at several stages. To test quality of BB fit, 15 consecutive EPID images were acquired without moving the BB and the standard deviation was calculated. To test the reproducibility of the imaging caliper system, 15 images were acquired, shifting the BB by 1.00 mm with caliper adjustment between images, and the mean and standard deviation of the difference between desired and calculated BB positions was calculated. Finally, end‐to‐end reproducibility was tested by performing seven consecutive measurements. In addition to comparing rotational errors, BB placement with respect to radiation isocenter was checked after measurement 4 and 7.

Four linac tables were measured: Linac‐A: Elekta Synergy S with HexaPod six degrees of freedom tabletop mounted on Elekta Precise table; Linac‐B and Linac‐C: Elekta Synergy with two‐piece Q‐Fix kVue tabletop (Qfix Systems, Avondale, PA) mounted on Elekta Precise table; Linac‐D: Elekta Precise with two‐piece Q‐Fix kVue tabletop mounted on Elekta Precise table. The Hexapod tabletop was parked for all measurements. Linac‐A was evaluated based on TG142 recommendations for stereotactic radiotherapy and Linac‐B, ‐C, and ‐D were evaluated as nonstereotactic machines.

## III. RESULTS

Table alignment was measured on four linacs with 49 measurements over 363 days. The four linacs had maximum table alignment error of 2.11, 1.88, 0.91, and 1.10 mm respectively (Linac‐A, ‐B, ‐C, and ‐D, respectively; Table 1).

**Figure 4:**
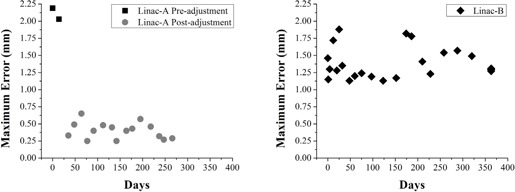
Maximum rotational error relative to MV isocenter over time. The Linac‐A table (left) before and after adjustment. The table exhibits stable performance for several months postadjustment. The Linac‐B table (right) does not exhibit significant trends from time of commissioning, but is characterized by greater standard deviation.

Linac‐A was shown to exceed the TG142 specification of ±1 mm from baseline for a stereotactic machine and was selected for adjustment using the proposed technique. Following adjustment to the screws at the table base, rotational error was significantly decreased to 0.40±0.12 mm (mean ± standard deviation, Fig. 4, p<0.001). This adjustment was stable over the course of 15 longitudinal measurements over the following 231 days (range 0.25—0.65 mm).

Linac‐B was tracked from time of commissioning with 27 measurements over 363 days. The alignment error was 1.40±0.22 mm (Fig. 4, range 1.13—1.88 mm). No significant trends in table behavior were observed after twelve months of clinical use. However, observed variability for the Linac‐B table was greater than variability for the Linac‐A table (standard deviation of 0.22 mm vs. 0.12 mm). This may be a result of the interchangeable two‐piece tabletop on Linac‐B, which allows more lateral movement than the one‐piece Linac‐A tabletop.

Reproducibility of the method was tested at several stages. For 15 consecutive EPID acquisitions without changing the position of the BB, the standard deviation of BB fit was 0.07 mm.

**Table 1:** Maximum rotational error measured on four linacs over 363 days

Measurements	*Linac‐A (preadjustment)* 2	*Linac‐A (preadjustment)* 15	*Linac‐B* 27	*Linac‐C* 2	*Linac‐D* 2
Mean (mm)	2.11	0.40	1.40	0.92	1.19
Range (mm)	2.03−2.19	0.25−0.65	1.13−1.88	0.91−0.92	1.10−1.28

**Figure 5:**
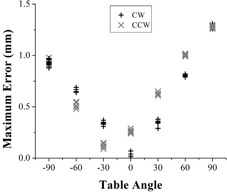
Calculated table misalignment is highly reproducible over seven sequential measurements, although different error profiles are observed with clockwise versus counterclockwise rotation.

For 15 consecutive measurements with known 1.00 mm shifts applied to the BB via calipers, the mean positional difference was 0.16 mm with standard deviation 0.05 mm (range 0.13‐0.21). Finally, end‐to‐end reproducibility was tested by performing seven consecutive measurements on Linac‐B (Fig. 5). Rotational error was highly reproducible at all table positions, with maximum standard deviation of 0.03 mm. Hysteresis in rotation was low, as final BB placement relative to radiation isocenter was within 0.13 mm of initial placement. However, different profiles were seen with clockwise and counterclockwise rotation.

## IV. DISCUSSION

The method described here provides for a semi‐automatic measurement of table rotation alignment that is accurate (on the order of 0.2 mm) and efficient (<1hr) using existing hardware and software. It furthermore provides the necessary adjustment to align the rotational axis of the table to radiation isocenter with submillimeter accuracy. Accurate table rotation is critical for highly conformal radiotherapy, especially noncoplanar methods. The rising interest in frame‐less stereotactic radiotherapy to body sites with image guidance has presented new challenges for quality assurance of linear accelerators. The requirement of placing high‐dose gradients in extreme proximity to critical structures, such as stereotactic radiotherapy of spine malignancies and the spinal cord,^20^, ^21^, ^22^, ^23^, ^24^, ^25^ has resulted in more stringent AAPM guidelines for treatment accuracy and the development of precision methods to characterize performance.^(26)^

In one study of spine stereotactic radiotherapy, translational errors of 2 mm, such as the ones found under rotational conditions in this study, resulted in dose error of greater than 5% to the target and 25% increase in dose to critical structures.^(27)^ In another study of spine stereotactic radiotherapy, simulated translational errors of 2 mm increased $D_{5}$ by up to 38%.^(28)^ The dosimetric consequences of table rotational error are a function of the number of noncoplanar treatment beams used, making it especially important for the proposed highly noncoplanar methods.^4^, ^5^, ^6^

The current standard for the alignment of linear accelerators, AAPM TG‐142, specifies a test of “coincidence of radiation and mechanical isocenter” on an annual basis.^(1)^ The tolerance is ±1 mm for units where stereotactic radiosurgery is performed. However, this tolerance is “from baseline”. Importantly, the report does not specify how this test may be accomplished, nor does it make recommendations of how to adjust the coincidence if it is out of tolerance. This issue is expected to be addressed in the upcoming AAPM TG‐198, which describes how to implement TG‐142 recommendations. The most commonly used measurements for isocenter coincidence, star‐shot and Winston‐Lutz tests, do not test alignment relative to radiation isocenter and do not provide the adjustment needed to minimize isocentric misalignment. The test developed here may be used for these purposes.

Other methods for measurement of table rotation have been described in the literature, including methods utilizing computerized analysis of film and EPID star‐shots, optical tracking, and phosphor plates.^9^, ^10^, ^11^, ^12^, ^13^, ^14^, ^15^, ^16^, ^17^, ^29^ The method we describe here differs from these previous studies in several important ways. First, the use of integrated linac systems allows efficient measurement in less than 1 hour and submillimeter accuracy without the need for external equipment. Some previous reported methodologies require specialized devices and/or time‐intensive procedures. Second, the rotational isocenter is characterized with knowledge of radiation isocenter. That is, while previous methods provide a means of measuring “wobble” as linac components are rotated,^15^, ^16^ the method presented here also links the wobble to the absolute position of the radiation isocenter in space. Third, the method uses a direct radiographic measure of radiation isocenter rather than relying on a surrogate, such as room lasers, which some methods rely on (e.g., Winkler at al.^(17)^). Finally, the proposed method provides the required adjustment to move the rotational axis to radiation isocenter, which can be achieved with greater precision than existing methods in the timeframe of a few hours.

Treatment table rotational performance showed no significant change over the course of 363 days, both for a table that received adjustment (Linac‐A) and a table that was monitored from the time of commissioning (Linac‐B). Note that, while Linac‐B exhibited submillimeter stability over time, the initial alignment at installation was relatively poor (over 1 mm). This makes this method well‐suited to adjust table rotation at acceptance testing, after an accidental collision or other deleterious event, or when starting a new SBRT treatment program. Variability in error was greater for Linac‐B than Linac‐A. We hypothesize that the single‐piece tabletop design of Linac‐A has reduced lateral motion under rotation versus the interlocking two‐piece tabletop of Linac‐B, as some lateral play exists in the locking bar.

Reproducibility of a single BB centroid fit over sequential measurements with known translations was less than 0.2 mm, limited by the accuracy of the Edmund rotary stages on the phantom, the EPID imaging panel, and the BB fitting methods. When averaged over 15 table positions, the end‐to‐end reproducibility of repeated rotational error assessment was less than 0.1 mm. It is notable that clockwise and counterclockwise rotation produced moderately distinct error profiles, most likely due to asymmetry in table braking characteristics. The stability of adjustment lends confidence to the use of this method.

The reader's attention is called to the fact that adjustment of the table base may propagate to other table characteristics, such as height, level, and optical reference frames, which should be evaluated after adjustment.

One limitation of this work is that it has only been tested on a single vendor platform. While we have used the reference BB method described above, in principle any method could be used which is capable of placing a radiographic object at, or close to, the radiation isocenter. Finally, the solution for determining radiation isocenter consists of only eight image acquisitions, and table rotation was only evaluated at 15 points. Increasing the number of images acquired could theoretically lead to better characterization of true mechanical and radiation isocenter coincidence. However, given the small standard deviation observed in the adjusted case, the implemented method appears sufficient to provide robust tracking of performance.

## V. CONCLUSIONS

The minimization of isocentric error is an important component of a comprehensive QA program. Dosimetric studies have demonstrated that relatively small misalignments (e.g., 2 mm) can lead to large dose errors (>25%) in some applications. A computerized method for precision measurement and adjustment of table rotation about radiation isocenter has been developed using a steel ball bearing and EPID images. Table rotation characteristics were shown to be stable over the course of twelve months on two linear accelerators, one of which was a new unit commissioned just prior to the tests performed here. The accuracy and efficiency of this method may make it suitable for acceptance testing, commissioning of highly conformal noncoplanar radiotherapy programs, and addition into an annual QA regimen.

## ACKNOWLEDGMENTS

We acknowledge useful discussions and assistance from Kevin Brown and Carlos Sandin (Elekta, Inc, Crawley, UK) and Erik Tryggestad, PhD (Mayo Clinic, Rochester, MN). The MATLAB code for this test is freely available upon request to the authors.
